# Robotic hepatectomy across IWATE difficulty levels: a single-center experience of the first 100 cases

**DOI:** 10.1007/s11701-026-03544-w

**Published:** 2026-06-08

**Authors:** Diego F. Chaparro-Zaraza, Jessica Lindemann, Amen Z. Kiani, Angela L. Hill, Neeta Vachharajani, Caleb Harsin, Darren R. Cullinan, William C. Chapman, Maria B. Majella-Doyle, Adeel S. Khan

**Affiliations:** Division of Abdominal Organ Transplantation, Department of Surgery, Washington University School of Medicine, 2660 South Euclid Avenue, Mailstop, Saint Louis, MO 8109-22-9905, 63110 USA

**Keywords:** Robotic liver resection, Hepatectomy, Minimally invasive surgery, IWATE criteria, Clavien-Dindo classification, Postoperative outcomes

## Abstract

This study describes our institutional experience with robotic hepatectomy, analyzes trends, and evaluates intraoperative and postoperative outcomes stratified by IWATE-defined procedural difficulty. Retrospective cohort study of the first 100 consecutive robotic hepatectomies performed by the abdominal transplant division at a high-volume center (2017–2025). Outcomes were compared across IWATE groups and between early (2017–2021) and late (2022–2025) cohorts. Logistic regression was used to examine the association between IWATE score and conversion to open surgery, 30-day complications, and major complications (Clavien–Dindo ≥ IIIa). Median age was 61 years (IQR 47–70), 52% were female, and 77% had malignant disease. 28% of resections were major. IWATE categories were low in 29%, intermediate 46%, advanced 7%, and expert 18%. Median operative time was 217 min (IQR 160–294), estimated blood loss was 150 mL (IQR 50–300), and length of stay was 3 days (IQR 2–4). Conversion to open surgery occurred in 22% with no emergent cases; 30-day complications occurred in 21%, major complications in 10%, readmission in 5% and 90-day mortality in 1%. Over time, expert cases and major hepatectomies increased without statistically significant differences in conversion or complication rates. Across IWATE groups, operative time, Pringle use, blood loss, transfusion, conversion, and length of stay increased with complexity (*p* < 0.001). Each 1-point increase in IWATE score independently increased the odds of conversion (adjusted OR 1.45, 95% CI 1.12–1.88) but not overall or major complications. Robotic hepatectomy was feasible across IWATE-defined difficulty levels while maintaining good perioperative outcomes. Increasing IWATE difficulty was associated with greater intraoperative complexity and a significantly higher likelihood of conversion to open surgery, although it was not independently associated with increased postoperative morbidity.

## Introduction

Minimally invasive liver surgery (MILS) has demonstrated significant benefits over open surgery, including lower blood loss, reduced transfusion requirements, shorter hospital stays, and fewer complication rates while maintaining comparable oncological outcomes [1–4]. Laparoscopic hepatectomy has gained widespread acceptance for minor resections, but its use in major or anatomically complex resections remains limited due to technical challenges and a steep learning curve [5–7].

Robotic liver surgery has emerged as a promising alternative, offering enhanced dexterity and precision, 3D visualization, and improved ergonomics. These features have facilitated the increasing adoption of a minimally invasive approach for more complex liver resections [8, 9].

The IWATE criteria provide a validated framework for objectively assessing the difficulty of laparoscopic liver resections. This system incorporates several factors including tumor location, size, proximity to major vessels, liver function, and the extent of the resection. Depending on these parameters, a score is assigned to the procedure performed, which classifies it into one of four levels of difficulty: low, intermediate, advanced, and expert [10]. Although originally designed for laparoscopic surgery, the IWATE system has become increasingly relevant in the emerging robotic era and has been validated as a tool for standardizing difficulty assessment in robotic liver surgery [11].

Currently, the relationship between IWATE-defined procedural difficulty and outcomes in robotic hepatectomy remains incompletely clarified. Some studies have found the IWATE score to be a significant predictor of operative time, blood loss, and length of stay, but not of postoperative complications in robotic liver resections [12, 13]. Others have suggested that the robotic platform may attenuate or even eliminate the increase in adverse outcomes observed with advanced and expert difficulty in laparoscopic hepatectomy [13–15]. In contrast, series focused on the implementation phase of robotic liver surgery have reported an increase in overall postoperative complications with higher IWATE scores, although major complications did not differ significantly across difficulty levels [16]. These heterogeneous findings highlight the need for further evaluation of how robotic hepatectomy performs across the full spectrum of IWATE-defined complexity.

This study aims to describe our initial 100-case experience with robotic hepatectomy, characterize temporal trends in case complexity, and evaluate intraoperative and postoperative outcomes stratified by IWATE-defined procedural difficulty.

## Materials and methods

### Study design and setting

This was a retrospective cohort study of the first one hundred consecutive patients who underwent robotic hepatectomy at our institution. Only cases performed by the Abdominal Transplant Surgery division were included. Transplant hepatectomies were excluded from the analysis. The procedures were performed by four surgeons with extensive training in hepatopancreatobiliary (HPB) and transplant surgery. Cases were completed over an 8-year period (January 2017 to May 2025). Institutional review board approval was obtained before initiation of the project (ID # 202504156).

### Robotic HPB and transplant surgery program

**Fig. 1:**
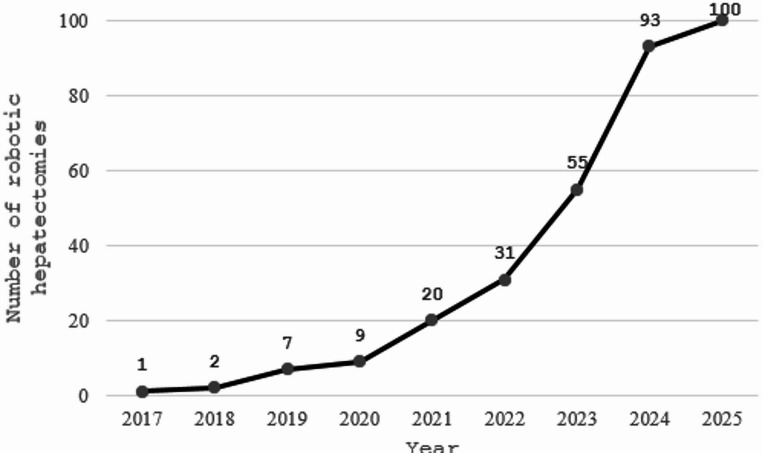
Growth in annual robotic hepatectomies between 2017 and 2025

**Fig. 2:**
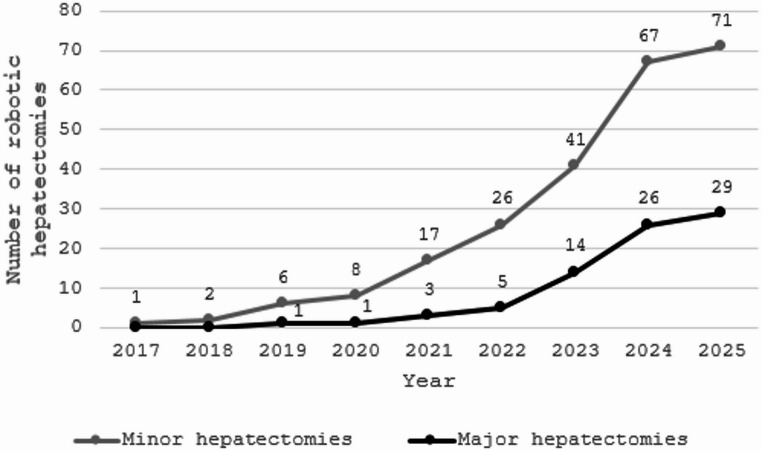
Annual number of minor and major robotic hepatectomies between 2017–2025

Robotic surgery was first introduced into our program in 2017 [17]. Initially, most procedures were low complexity HPB cases (e.g., simple cholecystectomy). In this same year, the first robotic minor hepatectomy, a resection of a biliary cystadenoma in segment 4 was accomplished. Less than a year later, in 2018, we performed the first robotic left lateral sectionectomy, and in 2019, our program completed the first major robotic liver resection which was a right hepatectomy with caudate lobe resection. In 2020, a dedicated robotic transplant (DaRT) team was incorporated into the OR team structure with the goal of providing intraoperative support to facilitate adoption of more complex HPB, living donor, and transplant operations. DaRT comprised first assists, scrub technicians and OR nurses with experience in transplant surgery who received additional training in all aspects of robotic surgery. Our aim was to gradually advance the complexity of procedures in line with the surgeons’ experience and confidence while maintaining patient safety as the top priority [17]. Figures 1 and 2 illustrate the growth in annual robotic hepatectomies during the study period.

### Patient selection

The robotic hepatectomy criteria have evolved over time. During the initial experience, most cases involved minor hepatectomies in patients with favorable body habitus (normal range of BMI, no prior abdominal surgeries) and tumors in favorable locations (segments 2, 3, 5 and 6). As experience increased, indications were expanded to patients requiring more complex resections (segments 4, 7 and 8), portal lymphadenectomy, and then to major hepatectomy. Currently, all patients requiring any hepatectomy in absence of vascular reconstruction are considered for robotic approach.

### Surgical technique

We use four 8 mm robotic trocars across the mid-abdomen with a 12 mm assistant port (Airseal) in the periumbilical or Pfannenstiel location which also serves as the specimen extraction site. Ports are adjusted higher or lower on the abdomen depending on the laterality of the resection (left vs. right) as well as the size of the liver. Intraoperative ultrasound (IOUS) is routinely used to define the transection margin and relevant vascular anatomy. For large resections, an intermittent Pringle maneuver is implemented using a foley catheter secured with a bulldog (Huang’s loop) around the porta hepatis. Parenchymal transection is performed using a combination of Monopolar and bipolar cautery, and Vessel Sealer (Intuitive Surgical, Inc., Sunnyvale, CA, USA). Large venous branches and crossing pedicles are controlled using Hem-o-lok clips and robotic or handheld staplers. At time of parenchymal transection during major hepatectomy, pneumoperitoneum pressure is often reduced, and PEEP increased to minimize risk of CO2 embolism. IOUS with duplex is routinely used to identify difficult to see tumors, assess relationships with major pedicles, and identification of landmarks (e.g. middle hepatic vein) to facilitate hepatic resection. Indocyanine green (ICG) is also routinely used to identify biliary anatomy, localize tumors, confirm transection planes, and to check for small bile leaks after parenchymal transection.

### Definitions and variables

For this study, all data were extracted from a prospectively maintained institutional database. Hepatectomies were classified according to the Brisbane 2000 terminology of liver anatomy and resection which defines major hepatectomy as the resection of three or more contiguous Couinaud liver segments [18]. Based on this definition, procedures were categorized as either major (right/left hepatectomy, extended right/left hepatectomy, and non-anatomic resections involving ≥ 3 segments) or minor (segmentectomy, left lateral sectionectomy, wedge resections involving < 3 segments, and radical cholecystectomy with segmental resection).

Surgical difficulty was stratified using the IWATE criteria, which scores resections from 0 to 12 based on tumor location, size, proximity to major vessels, extent of resection, and liver function. Cases were grouped as: Low (0–3), Intermediate (4–6), Advanced (7–9), and Expert (10–12) [10]. Continuous IWATE scores were used in regression analyses to evaluate the incremental association between increasing procedural difficulty and outcomes, whereas categorical IWATE groups (low, intermediate, advanced, and expert) were used for descriptive and comparative analyses to facilitate clinical interpretation and consistency with the standardized IWATE classification system.

Study variables included patient demographics, baseline clinical history, intraoperative data such as operative time, use of Pringle maneuver and its duration, conversion to open surgery, estimated blood loss, intraoperative transfusion, and intraoperative mortality. Postoperative outcomes included length of stay, need for postoperative transfusion, 30-day complications, 30-day readmission, 30-day and 90-day mortality. Complications were graded according to the Clavien-Dindo classification, with major complications defined as grade ≥3a. Postoperative bile leak and liver failure were graded according to the International Study Group of Liver Surgery criteria [19, 20].

Conversion to open surgery was classified into three groups according to timing and indication. Planned conversions were those in which it was decided preoperatively that the procedure would be started on the robotic platform and completed through an open approach. Urgent conversions included cases of unplanned intraoperative conversion necessary due to technical difficulty, inadequate exposure, or other reasons, but in the absence of an immediate life-threatening situation. Emergent conversions were unplanned conversions required for the management of a life-threatening complication such as uncontrolled bleeding, major vascular or biliary injury, or hemodynamic instability. The decision to convert was made intraoperatively at the discretion of the operating surgeon when continued robotic dissection was felt to compromise operative safety, oncologic adequacy, vascular control, or technical feasibility.

### Statistical analysis

Continuous variables were assessed for normality using histograms and the Shapiro–Wilk test and are reported as mean ± standard deviation or median (interquartile range [IQR]), as appropriate. Categorical variables are presented as counts and percentages. For comparisons across IWATE difficulty groups, categorical outcomes were compared using Chi-squared or Fisher’s exact tests, and continuous outcomes were compared using one-way ANOVA or the Kruskal–Wallis test for non-normally distributed data.

To evaluate temporal trends, cases were grouped into an early period (2017–2021) and a late period (2022–2025), corresponding to program initiation and subsequent expansion. IWATE scores, hepatectomy extension (major vs. minor), and key perioperative outcomes (Operative time, usage and duration of Pringle maneuver, conversion to open surgery, overall and 30-day major complication rates and length of stay), were compared between periods using Chi-squared or Fisher’s exact tests for categorical variables and the Wilcoxon rank-sum test for continuous variables.

Logistic regression was used to explore the relationship between IWATE score and key perioperative outcomes. Univariable models were first run with IWATE score (per 1-point increase) as the predictor for three endpoints: conversion to open surgery, any 30-day postoperative complications, and major 30-day postoperative complications (Clavien–Dindo grade ≥ 3). For conversion to open surgery and overall 30-day postoperative complications, a multivariable model was built including IWATE score, age, BMI, and extent of resection (major vs. minor hepatectomy). A multivariable regression model for 30-day major complications was not feasible because the number of events was small. For the same reason, and because several other outcomes were either continuous (and handled with non-parametric tests) or too infrequent, no additional multivariable models were performed. Results are presented as odds ratios (ORs) with 95% confidence intervals (CIs). All analyses were performed in Stata version 18 (Stata Corp LLC, College Station, TX), and a p value < 0.05 was considered statistically significant.

## Results

### Baseline characteristics

The median age for the cohort of 100 consecutive robotic hepatectomies was 61 years (IQR: 47–70), with 40% of patients 65 years or older. The majority were female (52%) and White (88%). The median BMI was 27.6 kg/m² (IQR: 23.9–32.5), with 37% classified as obese (BMI ≥ 30). Most patients had ASA III status (66%), and the most frequent comorbidities were cardiovascular (64%) and pulmonary disease (32%). Almost a third of patients (29%) had underlying liver disease, most commonly Metabolic Dysfunction Associated Steatohepatitis (MASH). Most patients (72%) had prior abdominal surgery. Malignancy was the most common indication for resection (77%), with colorectal liver metastases being the leading diagnosis. A detailed breakdown of baseline characteristics is presented in Table 1.

**Table 1:** Baseline patient characteristics (*n* = 100)

Variable	*n* (%) or Median (IQR)
*Age*,* years*	61 (47–70)
Age ≥ 65	40 (40)
Age ≥ 80	6 (6)
Age < 18	3 (3)
Female gender	52 (52%)
*Race*	
White	88 (88)
*BMI*, kg/m^2^	27.6 (23.9–32.5)
Obese (BMI ≥ 35)	37 (37)
Morbidly obese (BMI ≥ 40)	6 (6)
*ASA score*	
I	3 (3)
II	30 (30)
III	66 (66)
IV	1 (1)
*Comorbidities*	
Current smoker	21 (21)
Diabetes	24 (24)
Cardiovascular disease	64 (64)
Pulmonary disease	32 (32)
*Underlying liver disease*	29 (29)
MASH	19 (19)
Hepatitis B/C	6 (6)
Cirrhosis	4 (4)
*Prior abdominal surgery*	72 (72)
Open	27 (27)
Laparoscopic	28 (28)
Both	17 (17)
*Indication for resection*	
Benign	23 (23)
Malignant	77 (77)
Donor Hepatectomy	0 (0)
*Benign disease* (*n* = 23)	
Hepatic adenoma	9 (9)
Biliary cystadenoma	2 (2)
Hepatic cyst	2 (2)
Focal nodular hyperplasia	6 (6)
Hemangioma	2 (2)
Other symptomatic lesion	2 (2)
*Malignant disease* (*n* = 77)	
Hepatocellular carcinoma	16 (16)
Cholangiocarcinoma	13 (13)
Colorectal liver metastases	33 (33)
Gallbladder carcinoma	6 (6)
Other metastasis	9 (9)

### Intraoperative outcomes

The types of hepatectomy performed are detailed in Table 2. The median operative time was 217 min (IQR: 160–294). The Pringle maneuver was used in 40% of cases, with a median total clamping time of 37 min (IQR: 22–48). The overall conversion rate to open surgery was 22%; all cases were non-emergent categorized as either planned or unplanned (urgent). Estimated blood loss was 150 mL (IQR: 50–300), and 5% of patients required an intraoperative blood transfusion. No intraoperative deaths or complications occurred. Drains were placed in 83% of patients. Table 3 breaks down intraoperative outcomes of liver resections.

**Table 2:** Breakdown of robotic hepatectomies (*n* = 100)

Procedure type	*n* (%)
*Major hepatectomy*	28 (28)
Right hepatectomy	9 (9)
Extended right hepatectomy	4 (4)
Left hepatectomy	2 (2)
Extended left hepatectomy	4 (4)
Non-anatomic liver resection, ≥ 3 segments	8 (8)
Right posterior sectionectomy + anatomical segment 3 resection	1 (1)
*Minor hepatectomy*	72 (72)
Wedge/non-anatomic liver resection < 3 segments	44 (44)
Left lateral sectionectomy	21 (21)
Radical cholecystectomy w/seg 4b/5 resection and portal *Lymphadenectomy*	5 (5)
Segmentectomy (Single segment)	1 (1)
Right posterior sectionectomy	1 (1)

**Table 3:** Intraoperative outcomes for robotic hepatectomies (*n* = 100)

Variable	*N* (%) or median (IQR)
Operative time (minutes)	217 min (160–294)
*Pringle maneuver*	40 (40)
Duration of Pringle maneuver, minutes^a^	37 (22–48)
*Conversion to open surgery*	22 (22)
Emergent	0 (0)
Urgent	20 (20)
Planned	2 (2)
*Reason for conversion to open surgery* ^b^	
Complex anatomy/proximity to major structures	8 (36.4)
Limited exposure (liver/tumor size, adhesions or body habitus)	10 (45.4)
Bleeding risk concerns	2 (9.1)
Oncologic margin or tumor location concerns	2 (9.1)
Estimated blood loss, mL	150 (50–300)
*Need for intraoperative blood transfusion *	5 (5)
Major hepatectomy^b^	5 (17.2)
Minor hepatectomy	0 (0)
Intraoperative death	0 (0)
Drain placed	83 (83)

^a﻿^Among patients who received a Pringle maneuver

^b^Percentages are within that subgroup

### Postoperative outcomes

Table 4 details postoperative outcomes after robotic hepatectomy. The median length of hospital stay was 3 days (IQR: 2–4), The ICU requirement rate was 4%. During the 30-day postoperative period, major complications (Clavien-Dindo ≥ grade 3a) were documented in 10% of patients. Postoperative transfusion was required in 3% of patients, and the postoperative bile leak rate was 5%, with no grade C cases documented. No cases of postoperative liver failure were observed. The return to operating room rate was 1%. R0 resection was achieved in 87% of cases with a malignant diagnosis. The readmission rate was 5%. The 30-day mortality rate was 0%. One patient died in the 90-day postoperative period. This was a patient with a diagnosis of hepatocellular carcinoma who underwent a partial resection of segment six and afterwards chose to pursue hospice care.

**Table 4:** Postoperative outcomes after robotic hepatectomies (*n* = 100)

Variable	*N* (%) or median (IQR)
*Length of stay, days*	3 (2–4)
Major hepatectomy	4 (3–6)
Minor hepatectomy	2 (1–3)
Need for ICU admission	4 (4)
*ICU length of stay, daysᵃ*	0.22 (0–10)
Major hepatectomyᵃ	0.31 (0–7)
Minor hepatectomyᵃ	0.18 (0–10)
*Post-operative complications (Clavien-Dindo)*	21 (21)
1	5 (5)
2	6 (6)
3a	8 (8)
3b	0 (0)
4a	1 (1)
4b	1 (1)
5	0 (0)
*Bile leak*	5 (5)
A	3 (3)
B	2 (2)
C	0 (0)
Intra-abdominal infection	3 (3)
Surgical wound dehiscence	1 (1)
Myocardial infarction	1 (1)
Acute kidney injury	2 (2)
Pneumothorax	4 (4)
Urinary retention	1 (1)
Postoperative ileus	3 (3)
Need for post-operative Transfusion	3 (3)
*Resection margin*	
R0ᵇ	67 (87)
R1ᵇ	9 (13)
Return to operating room	1 (1)
*30-day readmission*	5 (5)
Major hepatectomy	2 (2)
Minor hepatectomy	3 (3)
*30-day mortality*	0 (0)
Major hepatectomy	0 (0)
Minor hepatectomy	0 (0)
*90-day mortality*	1 (1)
Major hepatectomy	0 (0)
Minor hepatectomy	1 (1)

ᵃReported as mean (min–max)

ᵇAmong patients with malignant disease

### Temporal trends

The distribution of IWATE difficulty groups differed significantly between periods. In the early period (2017–2021, *n* = 20), most cases were classified as low or intermediate difficulty, with no expert-level resections. In the late period (2022–2025, *n* = 80), expert cases accounted for 22.5% of procedures (*p* = 0.036). The proportion of major hepatectomies increased from 15.0% to 31.3%, although this difference was not statistically significant (*p* = 0.175). Median IWATE score rose from 4.5 (IQR 3–5) in the early period to 5 (IQR 3.5–9) in the later period, with a trend toward statistical significance (*p* = 0.070). Operative time remained comparable between periods [195 min (IQR 158–256) vs. 223 min (IQR 162–303.5), *p* = 0.233]. Pringle maneuver use increased from 15.0% to 46.3% (*p* = 0.011), and median clamping time also increased, although not significantly [13 min (IQR 5–56) vs. 39 min (IQR 24–47), *p* = 0.327]. Conversion to open surgery (20.0% vs. 22.5%, *p* = 1.000), estimated blood loss [100 mL (IQR 50–250) vs. 150 mL (IQR 62.5–300), *p* = 0.725], overall complication rate (20.0% vs. 21.3%, *p* = 1.000), and major complications (15.0% vs. 8.8%, *p* = 0.414) remained similar between periods, whereas median length of stay was modestly longer in the late period [2 days (IQR 1–3) vs. 3 days (IQR 2–4), *p* = 0.017]. When conversion rates were examined descriptively by IWATE category, low- and intermediate-difficulty resections in the early period had conversion rates of 11.1% each, whereas in the late period conversion rates were 5.0% and 8.1%, respectively. Advanced cases had conversion rates of 100% in the early period and 20.0% in the late period, while expert cases were present only in the late period, with a conversion rate of 72.2%. Because the early cohort was small and contained too few events, no further statistical comparisons by IWATE subgroup were performed.

### Intraoperative outcomes by IWATE difficulty

When stratified by IWATE difficulty, several intraoperative outcomes showed differences across groups (Table 5). Median operative time, usage of Pringle maneuver and estimated blood loss were higher for resections classified as advanced and expert (*p* < 0.001). The conversion rate rose from 6.9% in low difficulty cases and 8.7% in intermediate cases to 42.9% and 72.0% in advanced and expert resections, respectively (*p* < 0.001). Intraoperative transfusion showed a similar pattern, occurring only in advanced (14.3%) and expert cases (22.2%, *p* = 0.001). No intraoperative mortality or complications were observed in any group. In univariable logistic regression, each 1-point increase in IWATE score was associated with a 62% increase in the odds of conversion to open surgery (OR 1.62, 95% CI 1.32–1.98; *p* < 0.001), and this association remained significant after adjustment for age, BMI, and major versus minor hepatectomy (adjusted OR 1.45, 95% CI 1.12–1.88; *p* = 0.005) (Table 6).

**Table 5:** Intraoperative and postoperative outcomes stratified by IWATE preoperative difficulty

Outcome	Low (*n* = 29)	Intermediate (*n* = 46)	Advanced (*n* = 7)	Expert (*n* = 18)	*p* value
Operative time, min, median (IQR)	194 (133–237)	194 (160–241)	348 (312–448)	330 (271–388)	**< 0.001** ^a^
Pringle maneuver used	6 (20.7%)	14 (30.4%)	4 (57.1%)	16 (88.9%)	**< 0.001** ^c^
Duration of pringle, min, median (IQR)	40 (10–86)	33.5 (21–49)	48 (24–54.5)	33.5 (24–42.5)	**0.919** ^a^
Estimated blood loss, mL, median (IQR)	100 (50–200)	100 (50–200)	300 (150–500)	375 (250–630)	**< 0.001** ^a^
Conversion to open surgery	2 (6.9%)	4 (8.7%)	3 (42.9%)	13 (72.2%)	**< 0.001** ^c^
Intraoperative transfusion	0 (0%)	0 (0%)	1 (14.3%)	4 (22.2%)	**0.001** ^c^
Intraoperative complication^d^	0 (0%)	0 (0%)	0 (0%)	0 (0%)	N/A
Length of stay, days, median (IQR)	2 (1–3)	2 (2–4)	4 (3–8)	5 (4–6)	**< 0.001** ^a^
30-day overall complication rate	5 (17.2%)	6 (13.0%)	3 (42.9%)	7 (38.9%)	0.053^c^
30-day major complication rate^e^	4 (13.8%)	3 (6.5%)	1 (14.3%)	2 (11.1%)	0.578^c^
30-day mortality rate^d^	0 (0%)	0 (0%)	0 (0%)	0 (0%)	N/A
30-day readmission rate	2 (6.9%)	1 (2.2%)	0 (0%)	2 (11.1%)	0.329^c^
R0 resection^f^	22/23 (95.6%)	27/32 (84.4%)	5/6 (83.3%)	13/16 (81.3%)	0.466^b^

Data are presented as n (%) or median (IQR)

^a^Kruskal–Wallis test

^b^Chi-squared test

^c^Fisher’s exact test

^d^No intraoperative complications or 30-day deaths occurred in any group; statistical comparison not applicable

^e^Defined as Clavien–Dindo grade ≥ 3

^f^Denominator limited to patients with malignant disease

**Table 6:** Association between IWATE score and key perioperative outcomes (logistic regression)

Outcome	Model	OR per 1-point increase in IWATE	95% CI	*p* value
Conversion to open surgery	Univariable	1.62	1.32 1.98	**< 0.001**
Conversion to open surgery	Adjustedᵃ	1.45	1.12–1.88	**0.005**
30-day overall complication	Univariable	1.16	0.98–1.36	0.085
30-day overall complication	Adjustedᵃ	1.08	0.85–1.37	0.529
30-day major complicationᵇ	Univariable	0.93	0.72–1.19	0.540

ᵃAdjusted for age, BMI, and extent of resection (major vs. minor hepatectomy)

ᵇDefined as Clavien–Dindo grade ≥ 3

### Postoperative outcomes by IWATE difficulty

Length of stay was similar between low and intermediate cases but was significantly longer in advanced and expert resections (*p* < 0.001). Thirty-day postoperative morbidity also tended to be higher in more complex resections, although most differences did not reach statistical significance. Overall complication rates increased from 17.2% to 13.0% in low and intermediate cases to 42.9% and 38.9% in advanced and expert resections, respectively (*p* = 0.053). In univariable logistic regression, IWATE score showed a non-significant trend toward higher odds of any 30-day complication (OR 1.16 per 1-point increase, 95% CI 0.98–1.36; *p* = 0.085), and this association remained non-significant after adjustment for age, BMI, and major versus minor hepatectomy (adjusted OR 1.08, 95% CI 0.85–1.37; *p* = 0.529) (Table 6). Major complications occurred in 13.8%, 6.5%, 14.3%, and 11.1% of low, intermediate, advanced, and expert cases, respectively (*p* = 0.578), with no statistically significant association between IWATE score and major morbidity in univariable logistic regression (OR 0.93 per 1-point increase, 95% CI 0.72–1.19; *p* = 0.540) (Table 6). No multivariable logistic regression was performed for major complications due to the low volume of events. Thirty-day readmission rates ranged from 2.2% to 11.1% across groups (*p* = 0.329). R0 resection rates remained comparable across difficulty levels (95.6% low, 84.4% intermediate, 83.3% advanced, 81.3% expert; *p* = 0.466) (Table 5).

## Discussion

Over an eight-year period, our program experienced steady growth in robotic liver surgery, both in volume and complexity. In our early experience, most resections were of low or intermediate IWATE difficulty, with no expert-level cases and only a minority classified as major hepatectomies. In the more recent period, expert resections accounted for nearly one quarter of cases and the proportion of major hepatectomies more than doubled, reflecting growing confidence in using the robotic platform to treat patients with more complex disease. Importantly, this expansion was achieved without increasing conversion rates or perioperative morbidity. We believe a key factor in achieving this safe growth was the establishment of a dedicated robotic transplant team in 2020, increasing the number of robotic first assistants from zero to three and the number of surgeons trained in the robotic platform from one to four.

The overall perioperative profile of our cohort supports the growing evidence pointing out that robotic hepatectomy is a safe and feasible minimally invasive approach for benign and malignant disease. Our median operative time (217 min) was well within the range reported by other high-volume centers (145–321 min) [12–16, 21–28]. Median estimated blood loss (150 mL) and the intraoperative transfusion rate (5%) were likewise comparable and toward the favorable end of previously published series (EBL 30–321 mL [13, 14, 16, 21–24, 26, 27]; transfusion 1–16.9% [13, 23, 25–32]).

Our median length of stay (3 days) was similar to that reported in other robotic hepatectomy cohorts (3–6 days) [12–16, 21–24]. Overall 30-day complication (21%) and major complication (10%) rates fell within the ranges described in the literature (overall 7.4–34.5% [12–16, 23, 26, 28–32]; major 3.1–21% [12–16, 23, 24, 27–32]). The rate of R0 resection (87% of malignant cases) was also consistent with prior reports, where R0 margins range from approximately 76.6–99% depending on indication and extent of resection [2, 12, 13, 15, 21–25, 27–34]. Finally, our 30-day readmission rate (5%) and 90-day mortality (1%) were within the lower end of published ranges (readmission 3–17% [13, 14, 21–23, 25–31]; 90-day mortality 0–3% [14, 16, 22–26, 28, 31]).

Although our overall conversion rate (22%) was higher than that reported in prior series (0–11.3%) [2, 12–16, 21–31, 33, 34], it was largely concentrated in higher IWATE categories and major resections, with no emergent conversions, suggesting appropriate intraoperative judgment in the setting of high complexity rather than failure of the minimally invasive approach. Direct comparison across published series remains challenging due to substantial heterogeneity in patient selection, institutional experience, surgeon volume, tumor characteristics, and reporting methodology. In this early experience, the higher proportion of conversions may reflect the case mix and organizational structure at our institution, as a substantial number of robotic hepatectomies are performed by the surgical oncology division and were not captured in this cohort, which includes only cases performed by the abdominal transplant division. Additionally, this series included cases performed by four different surgeons and likely reflects the collective learning curve and ongoing standardization of robotic hepatectomy within the program. Previous studies using CUSUM analysis have estimated a learning curve for achieving proficiency in robotic hepatectomy of 28–75 cases for minor resections, 22–100 cases for major resections, and 54–57 for resections involving posterosuperior segments [22, 23, 35]. As the program matures and experience with complex robotic liver resections expands, we would expect conversion rates to decline, particularly in advanced and expert cases.

An additional descriptive observation supports this interpretation. Although the overall conversion rate remained stable over time, conversion patterns appeared to shift as the program matured. In the early period, low- and intermediate-difficulty resections each had conversion rates of 11.1%, whereas in the later period these fell to 5.0% and 8.1%, respectively. At the same time, conversions in the later era became increasingly concentrated in advanced and expert cases. This pattern suggests that as institutional experience increased, straightforward cases may have been completed robotically more consistently, while the stable overall conversion rate was driven by the deliberate expansion of the program toward more complex resections. Given the small size of the early cohort and limited number of events, this finding should be interpreted as descriptive rather than definitive.

Our study supports previous robotic hepatectomy series, which report that the IWATE-defined technical complexity is strongly associated with intraoperative parameters but less clearly linked to postoperative morbidity [12–15, 24]. As expected, higher IWATE categories correlated with longer operative times, increased use of inflow occlusion, greater blood loss, and a higher need for transfusions, with advanced and expert resections showing the highest resource utilization.

In our analysis, conversion to open surgery rose significantly across the spectrum of difficulty levels. In logistic regression, each 1-point increase in IWATE score independently raised the odds of conversion by approximately 45%, even after adjusting for age, BMI, and the extent of resection. This finding aligns with the initial experience reported by Toti et al. at the University of Chicago [13]. However, it is important to note that other published cohorts did not demonstrate such a clear gradient in conversion rates across difficulty groups. Some studies have reported relatively stable or uniformly low conversion rates despite increasing complexity, and others did not analyze conversion rates by difficulty category at all [12, 14–16, 21–25, 29]. Our data therefore may suggest that IWATE-defined complexity may be more closely linked to the likelihood of conversion than previously recognized, at least in the context of a maturing program.

In contrast, the relationship between IWATE-defined difficulty and postoperative outcomes was more nuanced. Although overall 30-day complication rates and length of stay tended to increase in advanced and expert cases, most differences in morbidity across IWATE groups did not reach statistical significance, and IWATE score was not independently associated with either overall or major complications in regression analyses. Importantly, complication rates were numerically higher in advanced and expert IWATE groups, suggesting that clinically meaningful differences may still exist and could become more apparent in larger cohorts. R0 resection rates were high and comparable, even in the most complex resections. Taken together, these observations suggest that, in our experience, increasing technical difficulty, as captured by the IWATE score, primarily manifested as a greater likelihood of conversion and modestly increased resource utilization, rather than a clear rise in postoperative morbidity. Difficulty scoring in robotic hepatectomy may help guide gradual case selection, operative planning, and patient counseling during program development, serving not only as a predictor of technical complexity but also as a practical framework for the structured and safe implementation of increasingly complex robotic liver resections.

This study has several strengths. It represents a consecutive series from a single transplant and HPB team, using a prospectively maintained database that captures detailed intraoperative and postoperative variables. Resections were classified according to the Brisbane 2000 terminology, technical difficulty was graded using the IWATE score, and complications were defined using the Clavien-Dindo classification and the Study Group of Liver Surgery criteria, allowing standardized reporting of resection extent, complexity, and morbidity. We also integrated both categorical IWATE groups and continuous IWATE scores into the analysis and used multivariable modeling to distinguish the effect of technical difficulty from that of patient and procedural characteristics. Nevertheless, there are limitations to be acknowledged. This study is a retrospective, single-center analysis conducted at a high-volume transplant and HPB center, which may limit generalizability and introduce selection bias. In addition, lower-complexity resections were preferentially performed earlier in the institutional experience, while advanced and expert IWATE cases were increasingly undertaken later in the maturation of the robotic hepatectomy program. As a result, procedural complexity, surgeon experience, patient selection, and institutional standardization are inherently intertwined, limiting causal interpretation of temporal and complexity-based comparisons. It is possible that the absence of a significant association between IWATE difficulty and postoperative morbidity partially reflects the mitigating effect of increasing institutional experience and improved perioperative workflows over time. The relatively small sample size, particularly for low-frequency outcomes such as major complications, limits statistical power and may increase the risk of type II error, potentially obscuring true associations between IWATE-defined difficulty and postoperative outcomes. The early cohort is smaller than the later cohort, making temporal comparisons more susceptible to random variation. Finally, this study focused on short-term perioperative outcomes, and long-term oncologic and functional outcomes were not evaluated and warrant further study.

## Conclusion

In this early single-center experience, robotic hepatectomy was feasible across a broad spectrum of IWATE-defined difficulty levels, including advanced and expert resections, while maintaining good perioperative outcomes. Increasing IWATE difficulty was associated with greater intraoperative complexity and a significantly higher likelihood of conversion to open surgery, although it was not independently associated with increased postoperative morbidity in this cohort. These findings support the use of the IWATE score as a practical tool for preoperative planning and suggest that, with appropriate patient selection and institutional expertise, higher technical complexity alone should not be viewed as a contraindication to a minimally invasive approach.

## Data Availability

The data are available upon request and with the permission of the Washington University School of Medicine in St. Louis.
